# The complete genome sequence, occurrence and host range of *Tomato mottle mosaic virus* Chinese isolate

**DOI:** 10.1186/s12985-016-0676-2

**Published:** 2017-01-31

**Authors:** Yueyue Li, Yang Wang, John Hu, Long Xiao, Guanlin Tan, Pingxiu Lan, Yong Liu, Fan Li

**Affiliations:** 1 Key Laboratory of Agro-Biodiversity and Pest Management of Education Ministry of China, Yunnan Agricultural University, Kunming, 650201 China; 2 Department of Plant and Environmental Protection Sciences, College of Tropical Agriculture and Human Resources, University of Hawaii, Honolulu, HI 96822 USA; 3 Modern Education Technology Center, Yunnan Agricultural University, Kunming, 650201 China; 4 Hunan Academy of Agricultural Sciences, Plant Protection Institute, Changsha, 410125 China

**Keywords:** *Tomato mottle mosaic virus*, Pepper, Complete genome sequence, *Tobamovirus*, Host range, Solanaceae, Cucurbitaceae

## Abstract

**Background:**

*Tomato mottle mosaic virus* (ToMMV) is a recently identified species in the genus *Tobamovirus* and was first reported from a greenhouse tomato sample collected in Mexico in 2013. In August 2013, ToMMV was detected on peppers (*Capsicum* spp.) in China. However, little is known about the molecular and biological characteristics of ToMMV.

**Methods:**

Reverse transcription-polymerase chain reaction (RT-PCR) and rapid identification of cDNA ends (RACE) were carried out to obtain the complete genomic sequences of ToMMV. Sap transmission was used to test the host range and pathogenicity of ToMMV.

**Results:**

The full-length genomes of two ToMMV isolates infecting peppers in Yunnan Province and Tibet Autonomous Region of China were determined and analyzed. The complete genomic sequences of both ToMMV isolates consisted of 6399 nucleotides and contained four open reading frames (ORFs) encoding 126, 183, 30 and 18 kDa proteins from the 5’ to 3’ end, respectively. Overall similarities of the ToMMV genome sequence to those of the other tobamoviruses available in GenBank ranged from 49.6% to 84.3%. Phylogenetic analyses of the sequences of full-genome nucleotide and the amino acids of its four proteins confirmed that ToMMV was most closely related to *Tomato mosaic virus* (ToMV). According to the genetic structure, host of origin and phylogenetic relationships, the available 32 tobamoviruses could be divided into at least eight subgroups based on the host plant family they infect: Solanaceae-, Brassicaceae-, Cactaceae-, Apocynaceae-, Cucurbitaceae-, Malvaceae-, Leguminosae-, and Passifloraceae-infecting subgroups. The detection of ToMMV on some solanaceous, cucurbitaceous, brassicaceous and leguminous plants in Yunnan Province and other few parts of China revealed ToMMV only occurred on peppers so far. However, the host range test results showed ToMMV could infect most of the tested solanaceous and cruciferous plants, and had a high affinity for the solanaceous plants.

**Conclusions:**

The complete nucleotide sequences of two Chinese ToMMV isolates from naturally infected peppers were verified. The tobamoviruses were divided into at least eight subgroups, with ToMMV belonging to the subgroup that infected plants in the Solanaceae. In China, ToMMV only occurred on peppers in the fields till now. ToMMV could infect the plants in family Solanaceae and Cucurbitaceae by sap transmission.

## Background

Peppers (*Capsicum* spp.) are economically important vegetable crops throughout the world. However, the increasing incidence and severity of virus infections in China recently has caused serious declines in yield and quality. More than 68 viruses have been reported infecting peppers in the world [1], including *Tobacco mosaic virus* (TMV), *Chilli veinal mottle virus* (ChiVMV), *Cucumber mosaic virus* (CMV), *Potato virus Y* (PVY), *Tomato spotted wilt virus* (TSWV), *Broad bean wilt virus 2* (BBWV2), *Tomato mosaic virus* (ToMV), *Pepper mild mottle virus* (PMMoV), and others. Four tobamoviruses were reported on peppers in China before 2014: TMV, ToMV, PMMoV and *Tobacco mild green mosaic virus* (TMGMV) [2–4]. *Tomato mottle mosaic virus* (ToMMV), a new species in the genus *Tobamovirus*, was recently identified infecting peppers in China in 2013 [5]. ToMMV was first identified on tomato in Mexico in 2013 [6], and subsequently was detected on tomatoes in the United States [7, 8], Israel [9] and Brazil (KT222999). Based on a comparison of partial nucleotide sequence similarities, ToMMV probably was present already in Iran (HQ593616, JX112024, JX112025, JX121570, JX121574, JX121575, JX121576), Brazil (AF411922, AM411425, AM411430) [10], and China (JX025564). These isolates, however, were identified as ToMV before ToMMV was characterized as a new *Tobamovirus* species. The isolate reported from Brazil (AF411922) might be the first sequence in the GenBank database corresponding to ToMMV [10].

*Tobamovirus* is the largest of six genera in the family Virgaviridae, it consists of 25 species and 6 tentative species, with TMV as the type species [11, 12]. According to the current online taxonomy released by ICTV (International Committee on Taxonomy of Viruses), however, there are now 35 members in the genus *Tobamovirus* (http://www.ictvonline.org/virusTaxonomy.asp). The known tobamovirus genomes contain four open reading frames (ORFs) encoding four proteins. The two larger polypeptides of 124–132 kDa and 181–189 kDa are involved in virus replication, the 124–132 kDa protein is terminated by an amber (UAG) stop codon and the 181–189 kDa protein is produced by a readthrough of this stop codon [13]. Two other ORFs encode the 28–31 kDa movement protein (MP) and the 17–18 kDa coat protein (CP). Tobamoviruses were previously divided into three subgroups based on the genomic structure and host range [13]. Min and co-workers [14] proposed that tobamoviruses could be divided into at least five subgroups according to the amino acid composition and primary structure of their CPs, and the hosts from which the viruses were originally isolated. Song et al. [15] suggested tobamoviruses should be divided into six subgroups based on the phylogenetic analysis of the four tobamovirus proteins with the existence of passifloraceae-infecting subgroup in the genus *Tobamovirus*.

There has been no detailed molecular information or biological characterization of ToMMV Chinese isolates until now, and no report about ToMMV infecting other plants except pepper and tomato. The genome sequence and genetic diversity analysis of ToMMV will help to characterize this emerging virus and develop appropriate detection methods. A better knowledge of the host range test of ToMMV will provide a theoretical basis for monitoring, prediction and effective prevention and control of the virus disease caused by ToMMV. This paper presented the complete genome sequences of two ToMMV isolates infecting peppers in China. The host range of ToMMV and the phylogeny of 32 available tobamoviruses were also analyzed.

## Methods

### ToMMV isolates and field investigation

ToMMV isolate YYMLJ (ToMMV-YYMLJ) with symptoms of foliar chlorosis, mosaic, and necrosis was collected from tabasco pepper (*Capsicum frutescens*) in Yuanmou County, Yunnan Province, China in 2013. ToMMV isolate TiLhaLJ (ToMMV-TiLhaLJ) was collected from bell pepper (*C. annuum* var. *grossum*) in Lhasa, Tibet Autonomous Region in China in 2013. Plants harboring this isolate had symptoms of foliar mottle, necrosis, and stunting.

To investigate the incidence and distribution of ToMMV, from 2013 to 2015, 1028 samples of symptomatic pepper (421), tomato (215), pumpkin (101), cucumber (17), radish (33), Chinese cabbage (48), kale (12), soybean (47), broad bean (46), cowpea (32), pea (8), and kidney bean (48) were collected from different fields in Yunnan Province, Tibet Autonomous Region, Hunan Province, Guizhou Province and Sichuan Province in China. The samples were tested by RT-PCR with ToMMV-specific primers ToMMVdF (CTGGAGAAGACTGGGTCTAG, identical to nt 4290–4309 of the ToMMV genome, GenBank Accession No. KF477193) and ToMMVdR (TTCGGTAAGTTCAATGGGACCT, complementary to nt 5482–5461). All samples were stored at −20 °C until processed. Total nucleic acids were extracted using a modified CTAB method [16] and processed separately. The extracts were stored at −20 °C.

### Host range test

To test the potential hosts of ToMMV, sap from ToMMV-positive pepper plants was ground in 0.1 M PBS buffer (pH 7.2) and mechanically inoculated onto *Nicotiana tabacum* var. Samsun at the 4- to 5-leaf stage. The inoculated tobacco plant showed systemic mosaic symptom but no local lesion was found. The inoculated plants was then detected with the specific primers of ToMMV, TMV, ToMV, PMMoV, TMGMV, CMV, TSWV, ToCV, BBWV2, and the degenerate primers of tobamovirus potyvirus, polerovirus and begomovirus by RT-PCR, and only ToMMV but no other virus was detected from the inoculated plants. So sap from inoculated *N. tabacum* var. Samsun with symptoms of infection was then mechanically inoculated onto the other 22 plants, including the solanaceous plants *Solanum lycopersicum*, *Capsicum annuum*, *N. benthamiana*, *N. tobacum*, *N. tabacum* var. Xanthi nc, *N. rustica*, *Nicandra physaloides*, *Physalis alkekengi*, *Petunia hybrida*, *Daturia stramonium*; the cruciferous plants *Brassica pekinensis*, *B. chinensis*, *Raphanus sativus*, *B. oleacea* var. *italica*, *B. oleracea* var. *botrytis*, *B. campestris*; the cucurbitaceous plants *Cucumis sativus*, *Cucurbita moschata*; the leguminous plants *Phaseolus vulgaris*, *Pisum sativum*, *Vigna unguiculata*, and others including *Ipomoea aquatica* in the Convolvulaceae.

### Amplification of the complete virus genome

The complete genome sequence of each ToMMV isolate was divided into six fragments and amplified by RT-PCR using designed primers. Each fragment was 1000- to 1500-bp long except the third segment, which was about 883 bp in length. There were overlapping areas between the adjacent two segments (Table 1). The six pairs of primers were designed according to the complete sequence of ToMMV (KF477193) accessed in GenBank. Reverse transcription amplification using Reverse Transcriptase M-MLV (RNase H^−^) (TaKaRa Biotech, Dalian, China), and PCR amplification using TaKaRa Ex Taq^TM^ (TaKaRa Biotech, Dalian, China) were conducted following the manufacturer’s instructions.

**Table 1:** Primers for complete genome amplification of ToMMV

Amplified fragments	sequence(5’-3’)	direction	site	length(bp)	Tm(°C)
ToMMV1	TTTAAGTATTTATTATTACAACAATTA	+	1 ~ 1465	1465	54.2
	GGACAGAAAGCTTTGTATGTAGG	-			
ToMMV2	TTTAAAGTCACCGCTAGGTCTGAGT	+	1377 ~ 2816	1440	60.5
	CCAGTGTGCAGCATCAATCC	-			
ToMMV3	GCTAAGGTTGTACTAGTAGACGG	+	2551 ~ 3433	883	60.5
	GTAATTGCTATTGAGTACCTGC	-			
ToMMV4	TTTAAACAGTCCCCATGTGCTTGTC	+	3313 ~ 4481	1209	60.6
	CCATCGGTAACATCGAGGCT	-			
ToMMV5	TTTAACTGGAGAAGACTGGGTCTAG	+	4290 ~ 5565	1193	60.3
	TTCGGTAAGTTCAATGGGACCT	-			
ToMMV6	TTTAAGGAACATATGGCAAGTCCTAGT	+	5311 ~ 6398	1089	60.5
	TGGGCCCCTACCGGGG	-			
5RAGSPR1	CAAGCGAGTGAACTGCGTTCTGAGTG	-	1 ~ 325	325	69.5
5RAGSPR2	GCCCGGGTAGCAATAAGTGTCTGTTC	-	1 ~ 269	269	69.5
3RAGSPF1	GTTAATGAATTGGTAAGAGGAACAGG TTTC	+	6106 ~ 6398	293	66.7

Annotation: TTTAA was added to the upstream primer according to the requirement of the cloning vector

To further verify the 5’- and 3’-end sequences of ToMMV, 5’-rapid amplification of cDNA ends (RACE) and 3’RACE were performed. 5’RACE was conducted with the SMART^TM^ RACE cDNA Amplification Kit (Clontech, USA) according to the manufacturer’s instructions. ToMMV-specific RACE Outer PCR primer 5RAGSPR1 (5’-CAAGCGAGTGAACTGCGTTCTGAGTG-3’) and SMART 5’RACE 10 × Universal Primer A Mix (long: 5’-CTAATACGACTCACTATAGGGCAAGCAGTGGTATCAACGCAGAGT-3’; short: 5’-CTAATACGACTCACTATAGGGC-3’) were used for Outer PCR amplification. ToMMV-specific RACE Inner PCR primer 5RAGSPR2 (5’-GCCCGGGTAGCAATAAGTGTCTGTTC-3’) and SMART 5’RACE Nested Universal Primer A (5’-AAGCAGTGGTATCAACGCAGAGT-3’) were used for Inner PCR amplification. 3’RACE was performed using 3’-Full RACE Core Set Ver.2.0 (TaKaRa Biotech, Dalian, China) with the ToMMV-specific primer 3RAGSPF1 (5’-GTTAATGAATTGGTAAGAGGAACAGGTTTC-3’) and 3’RACE Outer Primer (5’-TACCGTCGTTCCACTAGTGATTT-3’).

### Cloning and sequencing of viral genomic fragments

All amplified products were purified with a Universal DNA Purification Kit (TIANGEN Biotech, Beijing, China) and subsequently cloned into a pMD 19-T or pBackZero-T Vector (TaKaRa Biotech, Dalian, China) based on the manufacturers’ instructions. At least three independent clones from each ligation were sequenced on both strands (BGI Tech. Solutions, Shenzhen, China).

### Sequence assembly and analysis

The sequence data were assembled and analyzed with DNAstar 6.0 (DNAStar Inc, Madison,USA). The complete nucleotide sequences of ToMMV-YYMLJ and ToMMV-TiLhaLJ, as well as the deduced amino acid sequences of all four major proteins, were compared with the other 31 fully sequenced tobamoviruses available in GenBank (Table 2). Multiple sequence alignments were performed with ClustalW2 at http://www.ebi.ac.uk/Tools/msa/clustalw2/. Pairwise comparisons of the genome nucleotide sequence and the deduced protein sequence were performed among ToMMV and other members of the genus *Tobamovirus* by EMBOSS Pairwise Alignment Algorithms at http://www.ebi.ac.uk/Tools/msa/clustalo/. Phylogenetic relationships were analyzed by MEGA 6 [17] with aligned nucleotide or amino acid sequences of ToMMV and the other 31 tobamoviruses. Neighbor-joining trees were constructed using 1000 bootstrap replicates.

**Table 2:** Nucleotide sequence identity of the complete genome of ToMMV shared with other tobamoviruses

	ToMMV-TiLhaLJ	ToMMV-YYMLJ	ToMMV-MX5	ToMMV-10-100	ToMMV-NY-13
ToMV	84.2%	84.3%	84.3%	84.3%	84.3%
ToBRFV	80.6%	80.7%	80.8%	80.7%	80.8%
TMV	79.0%	79.1%	79.0%	79.1%	79.1%
ReMV	78.0%	78.1%	78.4%	78.4%	78.4%
BPMoV	76.2%	76.3%	76.4%	76.4%	76.6%
PMMoV	69.0%	69.0%	68.9%	69.1%	69.0%
BrMMV	66.2%	66.1%	65.9%	66.3%	66.1%
TMGMV	65.6%	65.6%	65.5%	65.5%	65.4%
YTMMV	64.6%	64.6%	64.6%	64.6%	64.7%
ObPV	64.5%	64.5%	64.6%	64.6%	64.4%
PaMMV	64.3%	64.3%	64.4%	64.4%	64.2%
ORSV	59.4%	59.5%	59.5%	59.2%	59.3%
RMV	59.2%	59.2%	59.3%	59.2%	59.2%
YMoV	59.4%	59.4%	58.7%	58.7%	58.9%
WMoV	58.6%	58.7%	58.9%	58.7%	58.8%
TVCV	58.4%	58.4%	58.5%	58.6%	58.5%
SFBV	58.0%	58.0%	58.1%	57.9%	58.2%
HLSV	53.2%	53.4%	53.1%	52.7%	52.8%
HLFPV	52.4%	52.3%	52.3%	52.3%	52.1%
FrMV	51.8%	52.1%	52.1%	51.9%	52.0%
CGMMV	52.0%	52.0%	51.8%	51.9%	52.0%
RCNaV	52.0%	52.0%	52.1%	52.1%	51.9%
CuMoV	51.4%	51.5%	51.4%	51.5%	51.5%
CYMV	51.0%	51.0%	51.2%	51.4%	51.1%
SHMV	50.8%	50.6%	50.6%	50.4%	50.7%
KGMMV	50.1%	50.1%	50.3%	50.4%	50.3%
ZGMMV	50.0%	50.2%	50.2%	50.2%	50.2%
CMMoV	50.6%	50.2%	50.5%	50.1%	50.2%
MarMV	50.2%	50.1%	50.2%	50.3%	50.1%
PafMV	50.1%	50.1%	50.0%	49.9%	50.1%
CFMMV	50.1%	49.8%	49.8%	49.6%	49.8%

## Results

### Genome organization of ToMMV

**Fig. 1:**
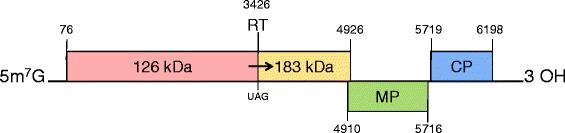
Genome organization of ToMMV-YYMLJ and ToMMV-TiLhaLJ. Numbers in small font indicate genomic positions of each ORF (numbered in large font). The 183 kDa ORF is the read-through region from the 126 kDa ORF; MP = movement protein; CP = coat protein; 5m^7^G = 7 methyl guanine nucleoside at the 5’ end; UAG = the amber codon; RT = read through

The complete genome sequences of the two ToMMV isolates (YYMLJ, Acc. No. KR824950; TiLhaLJ, Acc. No. KR824951) from peppers in China were the same length and had the same genomic structure as three other ToMMV tomato isolates: MX5 (KF477193) from Mexico [6], 10–100 (KP202857) [7] and NY-13 (KT810183) from the United States. The genomes of YYMLJ, TiLhaLJ and 10–100 consisted of 6399 nucleotides (nt) with an additional “A” at nt 6212, compared with MX5 and NY-13, and encoded four ORFs. The 5’ untranslated region (UTR) was 75nts long with a so-called “Ω fragment” that had no G residue except the m^7^G cap at the 5’ ultimate nucleotide [18, 19]. The 3’ UTR was 201nts long with a conserved region ^6,271^TCCCTCCACTTAAATCGAAGGGTT^6,294^ ending with the sequence CCCA typical of other tobamoviruses [20]. ORF1 started at nt 76 and encoded a putative protein of 126 kDa, and the 183 kDa readthrough protein started from a leaky UAG stop codon at nt 3426 and terminated at nt 4926 with a 54 kDa polypeptide in the readthrough region. The third ORF encoded a 30 kDa MP from nt 4910 to nt 5716, and ORF4 extended between nt 5719–6198 with an intergenic region of 2nts between the MP and CP ORFs, resulting in a 18 kDa CP (Fig. 1).

### Whole genome alignment and analysis of ToMMV compared with other tobamoviruses

The full-length nucleotide sequences of available ToMMV isolates were compared with each other and with the 31 other tobamoviruses. The genomes of ToMMV-YYMLJ and ToMMV-TiLhaLJ shared identities of 99.9% at the nucleotide sequence level, so the genomic sequence of ToMMV-YYMLJ was therefore used in subsequent sequence analyses. ToMMV-YYMLJ shared full-length nucleotide sequence identities of 99.4%–99.5% with ToMMV-NY-13, ToMMV-MX5 and ToMMV-10-100. The five ToMMV isolates were 49.6%–84.3% homologous to the other 31 tobamoviruses. They shared the highest identities of 84.3% and 80.8% with ToMV and Tomato brown rugose fruit virus (ToBRFV) respectively, and less than 80% homology with the other 29 tobamoviruses (Table 2). The amino acid (aa) sequences of 126 kDa, 54 kDa, MP and CP of ToMMV respectively displayed 38.1%–94.4%, 47.6%–96.2%, 17.4%–80.3% and 21.2%–91.2% identity to those of the other 31 tobamoviruses.

Gibbs et al. [21] reported a genus-specific nucleotide motif for tobamoviruses, the “4404-50 motif ”, twenty nine sites of this 47nts sequence are invariant in all tobamovirus sequences. Eighteen of the sites in the 4404–50 motif varied between species, but at 12 or more of these sites all isolates of each species usually had the same nucleotide [21]. Multiple sequence alignments revealed that the ToMMV genome also contains the distinctive 4404–50 motif of ^4411^GGTGATGTTACAACTTTCATAGGAAATACTGTTATTATAGCCGCGTG^4457^ (underlined bases are different with other tobamoviruses). All of the 18 variable sites are invariant among the 5 ToMMV isolates, while the conserved nucleotides in the ToMMV sequences differ from those in ToMV at 9 of the sites, which clearly distinguishes ToMMV from ToMV as follows: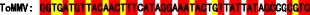 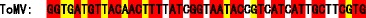


Red-shaded sites were the same in all tobamoviruses, yellow-shaded sites were the same in ToMMV and ToMV, and the unshaded sites varied among different isolates of ToMMV and ToMV. No more than 9 of the 18 conserved sites of ToMMV were shared with other tobamoviruses. The results of these comparisons strongly confirmed ToMMV as a distinct member in the genus *Tobamovirus*.

### Phylogenetic relationship of ToMMV with other tobamoviruses

**Fig. 2:**
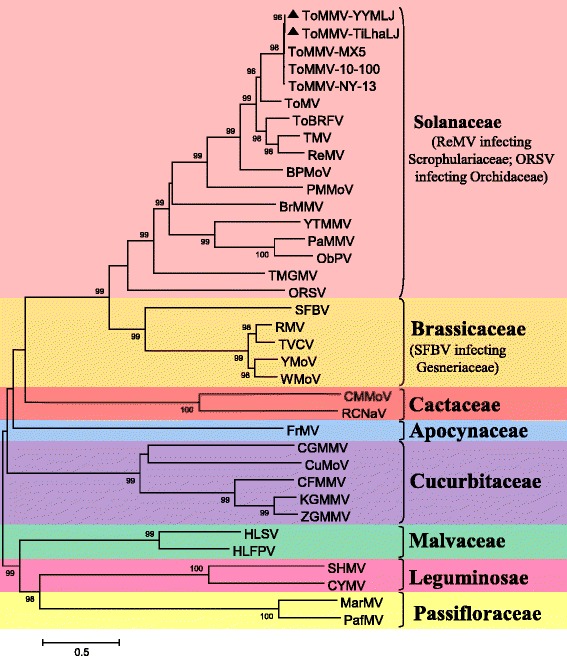
Phylogenetic analysis of ToMMV and members of the genus *Tobamovirus* based on their complete nucleotide sequences. The phylogenetic tree was constructed by the neighbor-joining algorithm using the MEGA 6 and subjected to 1000 bootstrap replicates. The following sequences of tobamoviruses were obtained from the GenBank database for comparisons: BPMoV, DQ355023; BrMMV, AM398436; CFMMV, AF321057; CGMMV, D12505; CMMoV, EU043335; CuMoV, AB261167; CYMV, JN566124; FrMV, HM026454; HLFPV, AB917427; HLSV, AF395898; KGMMV, AJ295948; MarMV, DQ356949; ObPV, D13438; ORSV, X82130; PafMV, HQ389540; PaMMV, AB089381; PMMoV, M81413; RCNaV, JF729471; ReMV, EF375551; RMV, GQ401365; SFBV, AM040955; SHMV, HH820921; TMGMV, JX534224; TMV, V01408; ToBRFV, KT383474; ToMMV-10-100, KP202857; ToMMV-MX5, KF477193; ToMMV-NY-13, KT810183; ToMMV-TiLhaLJ, KR824951; ToMMV-YYMLJ, KR824950; ToMV, AF332868; TVCV, U03387; WMoV, AB017504; YMoV, U30944; YTMMV, KF495565; ZGMMV, AJ295949

**Fig. 3:**
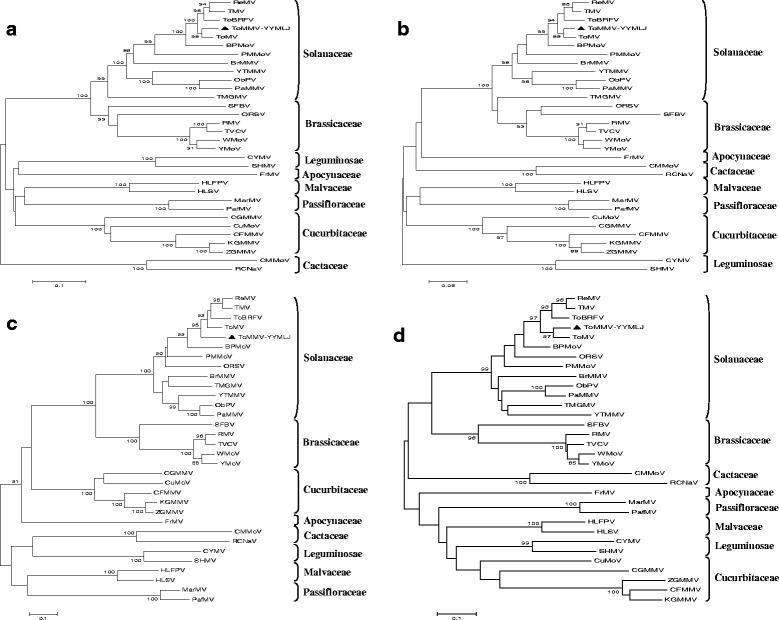
Phylogenetic analysis of ToMMV and members of the genus *Tobamovirus* based on the amino acid sequences of the 126 kDa replicase (**a**), 54 kDa polymerase (**b**), 30 kDa MP (**c**) and 18 kDa CP (**d**). Phylogenetic trees were constructed by the neighbor-joining algorithm using the MEGA 6 and subjected to 1000 bootstrap replicates. ReMV infecting the Scrophulariaceae, ORSV infecting the Orchidaceae and SFBV infecting the Gesneriaceae

The phylogenetic tree analyses based on the complete genome and the 126 kDa, 54 kDa, MP and CP amino acid sequences both showed ToMMV grouped with ToMV, ToBRFV, TMV and other tobamoviruses infecting solanaceous plants, and ToMMV was most closely related to ToMV (Figs. 2 and 3). The phylogenetic analyses and the host from which the viruses were originally isolated showed the currently available 32 tobamoviruses could be divided into at least eight subgroups according to their host-plant families: Solanaceae-, Brassicaceae-, Cactaceae-, Apocynaceae-, Cucurbitaceae-, Malvaceae-, Leguminosae-, and Passifloraceae-infecting subgroups. This grouping was based on the complete nucleotide sequences (Fig. 2) and the amino acid sequences of the 126 kDa replicase (Fig. 3a), 54 kDa polymerase (Fig. 3b), MP (Fig. 3c) and CP (Fig. 3d).

### Occurrence and the host range of ToMMV

From 2013 to 2015, ToMMV was only detected on peppers from Yuanmou and Mengzi counties of Yunnan Province, Lhasa in the Tibet Autonomous Region and Changsha in Hunan Province. No ToMMV was detected from these plants with virus-like symptoms: tomato, pumpkin, cucumber, radish, Chinese cabbage, kale, soybean, broad bean, cowpea, pea, and kidney bean. The overall incidence of naturally infected pepper was 3.56%. Most of the infected peppers showed the symptoms of foliar mottle and mosaic.

**Fig. 4:**
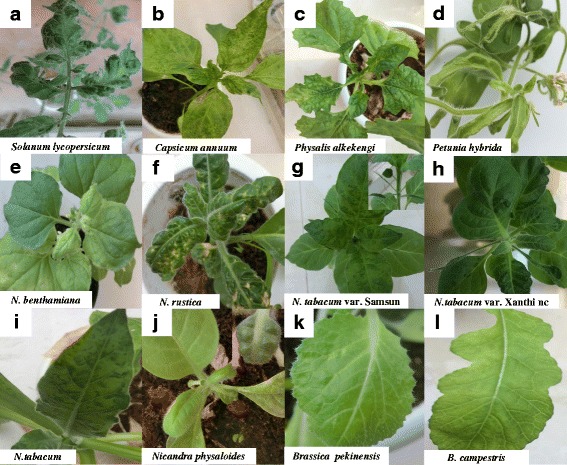
Symptoms on the tested plants inoculated with ToMMV. **a** Mosaic, blistering and distortion on *Solanum lycopersicum*. **b** Mosaic and necrosis on *Capsicum annuum*. **c** Mosaic and chlorosis on *Physalis alkekengi*. **d** Mosaic, distortion and leaf narrowing on *Petunia hybrida*. **e** Chlorosis on *Nicotiana benthamiana*. **f** Blistering, distortion and necrosis on *N. rustica*. **g** Mosaic, blistering and leaf narrowing on *N. tabacum* var. Samsun. **h** Mosaic, blistering, distortion and leaf narrowing on *N. tabacum* var. Xanthi nc. **i** Mosaic on *N. tabacum*. **j** Mottle, blistering and distortion on *Nicandra physaloides*. **k** Mottle on *Brassica pekinensis*. **l** Mottle on *Brassica campestris*

ToMMV infected 16 of 23 plants by mechanical transmission, including *N. tabacum* var. Samsun, *Solanum lycopersicum*, *Capsicum annuum*, *N. benthamiana*, *N. tabacum*, *N. tabacum* var. Xanthi nc, *N. rustica*c, *Nicandra physaloides*, *Physalis alkekengi* and *Petunia hybrida* in the family Solanaceae; *B. pekinensis*, *B. chinensis*, *Raphanus sativus*, *B. oleacea* var. *italica*, *B. oleracea* var. *botrytis* and *B. campestris* in the family Brassicaceae. These infected plants were also detected by RT-PCR with the specific primers of ToMMV, TMV, ToMV, PMMoV, TMGMV, CMV, TSWV, ToCV, BBWV2, and the degenerate primers of tobamovirus, potyvirus, polerovirus and begomovirus, and only ToMMV was detected. The virus caused systemic symptoms including mosaic (Fig. 4a, b, c, d, g, h, i), blistering (Fig. 4a, f, g, h, j) and chlorosis (Fig. 4c, e) on the majority of infected species with occasionally severe foliar distortion (Fig. 4a, d, f, h, j), leaf narrowing (Fig. 4d, g, h) and necrosis (Fig. 4b, f) in the infected solanaceous plants, while the virus caused mottle symptom in the infected *B. pekinensis* (Fig. 4k), *B. campestris* (Fig. 4l), *B. chinensis*, and *Raphanus sativus*, but no distinct symptom in *B. oleacea* var. *italica* and *B. oleracea* var. *botrytis*. ToMMV infection was confirmed by RT-PCR in these plants, and the results revealed ToMMV may be one of the main viruses infecting the solanaceous plants. The host plant species and their symptoms caused by ToMMV were confirmed by agro-infiltration with the infectious clone of ToMMV (data not shown).

## Discussion

ToMMV host range test of 23 plants species in 5 families suggested the pathogenicity of ToMMV might be limited to the Solanaceae and Brassicaceae. This was a narrow sampling of plant species. Worthy to notify, some infections previously identified as ToMV may have been ToMMV, including those on *Solanum lycopersicum* (AF411922, JX025564, JX112024, JX112025, JX121575, JX121576, KF477193, KP202857, KT810183), *Capsicum* spp. (AM411425, AM411430, JX121574, KJ605653), *Phaseolus vulgaris* (JX121570), and *Chenopodium murale* (HQ593616). Therefore, more plants are required to be tested for the host range of ToMMV, and additional surveys are necessary to determine the global distribution of this emerging virus.

Min et al. [14] proposed dividing the tobamoviruses into at least five subgroups according to their amino acid composition and primary structure of their CPs, these virus were originally isolated from plants in the Solanaceae, Brassicaceae, Cucurbitaceae, Cactaceae and Malvaceae. Song et al. [15] suggested the existence of a sixth subgroup in the genus *Tobamovirus* isolated from the Passifloraceae, based on the phylogenetic analysis of the four tobamovirus proteins. Here, according to the phylogenetic analyses and the hosts from which the viruses were originally isolated, the presently available 32 tobamoviruses could be divided into at least eight subgroups, Solanaceae-, Brassicaceae-, Cactaceae-, Apocynaceae-, Cucurbitaceae-, Malvaceae-, Leguminosae-, and Passifloraceae-infecting subgroups based on both the complete nucleotide sequences and 126 kDa, 54 kDa, MP and CP amino acid sequences. In addition, those tobamoviruses infecting plants in the Solanaceae were most closely related to the tobamoviruses infecting plants in the Brassicaceae, which was consistent with the results of host range test.

*Ribgrass mosaic virus* (ReMV) which infecting the Scrophulariaceae was always clustered with the solanaceae-infecting subgroup, while *Streptocarpus flower break virus* (SFBV) which infecting the Gesneriaceae was grouped into the cruciferae-infecting subgroup no matter at the nt or the aa sequence levels (Figs. 2 & 3). Whereas *Odontoglossum ringspot virus* (ORSV) which infecting the Orchidaceae was divided into the solanaceae-infecting subgroup based on the nt and aa sequences of its MP and CP, but clustered with the brassica-infecting subgroup based on the aa sequences of its 126 kDa replicase and 54 kDa polymerase (Figs. 2 & 3). ToMMV grouped with ToMV, ToBRFV, TMV and other tobamoviruses infecting solanaceous plants, and ToMMV was most closely related to ToMV (Figs. 2 & 3). Both our phylogenetic tree analyses of the complete genome and the 126 kDa, 54 kDa, MP and CP amino acid sequences strongly supported that ToMMV belongs to the subgroup of the tobamoviruses that infects plants in the Solanaceae.

More surveys are needed to determine the incidence and distribution of ToMMV in the field. Research to identify genetic mechanisms of pathogenesis and host-plant defense will assist in the development of crop resistance to ToMMV.

## Conclusions

The complete sequence of ToMMV Chinese isolates from pepper (*Capsium* spp.) and phylogenetic relationship among ToMMV and other tobamoviruses were described in this study. The tobamoviruses could be divided into at least eight subgroups based on the plant families they infected: Solanaceae-, Brassicaceae-, Cactaceae-, Apocynaceae-, Cucurbitaceae-, Malvaceae-, Leguminosae-, and Passifloraceae-infecting subgroups. All our results indicated that ToMMV belonged to the solanaceae-infecting subgroup in the genus *Tobamovius*, and the tobamoviruses that infected plants in the Solanaceae were most closely related to the tobamoviruses that infected plants in the Brassicaceae. ToMMV occurred on peppers in the fields of Yunnan Province, Hunan Province and Tibet Autonomous Region of China. ToMMV could infect plants in the Solanaceae and Brassicaceae by sap transmission.

## Abbreviations

BBWV2: *Broad bean wilt virus 2*
BPMoV: *Bell pepper mottle virus*
BrMMV: *Brugmansia mild mottle virus*
CFMMV: *Cucumber fruit mottle mosaic virus*
CGMMV: *Cucumber green mottle mosaic virus*
ChiVMV: *Chilli veinal mottle virus*
CMMoV: *Cactus mild mottle virus*
CMV: *Cucumber mosaic virus*
CP: Coat protein
CuMoV: *Cucumber mottle virus*
CYMV: *Clitoria yellow mottle virus*
FrMV: *Frangipani mosaic virus*
HLFPV: *Hibiscus latent Fort Pierce virus*
HLSV: *Hibiscus latent Singapore virus*
ICTV: International committee on taxonomy of viruses
KGMMV: *Kyuri green mottle mosaic virus*
MarMV: *Maracuja mosaic virus*
MP: Movement protein
ObPV: *Obuda pepper virus*
ORFs: Open reading frames
ORSV: *Odontoglossum ringspot virus*
PafMV: *Passion fruit mosaic virus*
PaMMV: *Paprika mild mottle virus*
PBS: Phosphate Buffer Saline
PCR: Polymerase chain reaction
PMMoV: *Pepper mild mottle virus*
PVY: *Potato virus Y*
RACE: Rapid amplification of cDNA ends
RCNaV: *Rattail cactus necrosis*-*associated virus*
ReMV: *Rehmannia mosaic virus*
RMV: *Ribgrass mosaic virus*
RT-PCR: Reverse transcriptase polymerase chain reaction
SFBV: *Streptocarpus flower break virus*
SHMV: *Sunn*-*hemp mosaic virus*
TMGMV: *Tobacco mild green mosaic virus*
TMV: *Tobacco mosaic virus*
ToBRFV: Tomato brown rugose fruit virus
ToMMV: *Tomato mottle mosaic virus*
ToMV: *Tomato mosaic virus*
TSWV: *Tomato spotted wilt virus*
TVCV: *Turnip vein*-*clearing virus*
UTR: Untranslated region
WMoV: *Wasabi mottle virus*
YMoV: *Youcai mosaic virus*
YTMMV: *Yellow tailflower mild mottle virus*
ZGMMV: *Zucchini green mottle mosaic virus*.
